# Transient fixation of L4 vertebra preserves lumbar motion and function in Lenke Type 5C and 6C scoliosis

**DOI:** 10.1038/s41598-021-89674-7

**Published:** 2021-05-13

**Authors:** Jae-Ho Yang, Jae-Won Shin, Sub-Ri Park, Sun-Kyu Kim, Sang-Jun Park, Ji-Hwan Min, Byoung-Ho Lee, Kyung-Soo Suk, Jin-Oh Park, Seong-Hwan Moon, Hwan-Mo Lee, Hak-Sun Kim

**Affiliations:** 1 Department of Orthopedic Surgery, Yonsei University College of Medicine, 50 Yonsei-ro, Seodaemun-gu, Seoul, 03722 Korea; 2 Department of Orthopaedic Surgery, Gangnam Severance Hospital, Yonsei University College of Medicine, 211Eonju-ro, Gangnam-gu, Seoul, 06273 Korea

**Keywords:** Musculoskeletal system, Outcomes research, Diseases

## Abstract

This study investigated the efficacy of a novel surgical method that relies on the transient fixation of L4 in Lenke Type 5C and 6C adolescent idiopathic scoliosis. Thirty-six transient surgically treated L4 fixation patients were retrospectively evaluated. The first surgery involved mechanical correction of scoliosis; the lowest instrumented vertebra (LIV) was L4. After an average of 1.3 years (range, 0.3–3.4), the second surgery to remove transient L4 pedicle screws was performed. Radiographic parameters and SRS-22 scores were measured. Cobb’s angle, coronal balance, LIV tilting angle, and LIV coronal disc angle clearly improved after the first surgery (p < 0.01). After the second surgery, the corrected Cobb angle (p = 0.446) and coronal balance were maintained (p = 0.271). Although L3/S1 lumbar lordosis decreased after the first surgery (p < 0.01), after removal of transient L4 pedicle screws, it recovered slightly (p = 0.03). Similarly, the preoperative L3/4 lateral disc mobility eventually recovered after transient L4 screw removal (p < 0.01). The function domain of the SRS-22 showed better scores after removal of transient L4 screws (p = 0.04). L4 transient fixation surgery is beneficial for Lenke Type 5C and 6C scolioses that do not fully satisfy LIV (L3) criteria. It preserves L3/4 disc motion, increases functional outcomes, and maintains spinal correction and coronal balance.

## Introduction

The surgical treatment of scoliosis was first reported in 1924 by Russell Hibbs ^1^. Since then, many studies have been conducted investigating the nature of the idiopathic scoliosis curve. In 1962, the development of a metal vertebral fixation device by Harrington significantly altered scoliosis treatment ^2^. Subsequently, the King and Moe classification system was used to identify spine curvature types and allowed surgeons to determine a fusion range during spinal surgery ^3,4^. In the 1980s, following an introduction of thoracic pedicle screw system, a new classification for idiopathic scoliosis was developed by Lenke et al. ^5^, which attempted to standardize and minimize fusion levels. However, for Lenke Types 5C and 6C scolioses, the optimal distal fixation level remains debatable.

Scoliosis requires a relatively long level of fusion. Thus, excessive pressure is applied to the distal level disc. Reduction of one distal fusion level in the lumbar spine might be crucial for better long-term outcomes. However, in Lenke Types 5C and 6C, the distal fusion level is set to L3 or L4, despite the lack of accepted standards. Lenke insisted that if patients met the following criteria, only L3 could be fixed: (1) less than grade I rotation of L3, (2) L3 tilt < 30° and L4 tilt < 20°, (3) bisected L4 vertebral body from the center of the sacrum vertical line (CSVL), (4) apical disc located above L1-L2, (5) parallel L3-L4 opening or opposite of the L4-L5 disc level, and (6) centered location of L3 ^6^. However, it is too complicated to apply these six criteria, so other authors emphasized only two of them: (1) the degree of L3 rotation is below the Nash-Moe Grade II, and (2) L3 is the stable vertebra on bending radiographs ^7^. Wang et al. ^8^ suggested that L3 should be a distal fusion level; (1) if L3 presents < 28-mm translation and (2) if L3 exhibits < 25° tilt. These overwhelming variations in L3 fixation criteria negatively impact the decision-making process of the distal fusion level in surgical treatment for idiopathic scoliosis in actual clinical practice. This study aimed to introduce a novel surgical method of transient fixation up to the L4 vertebrae for non-canonical scoliosis patients who do not fully meet one of the aforementioned criteria in Lenke Types 5C and 6C.

## Results

### Demographics

**Figure 1:**
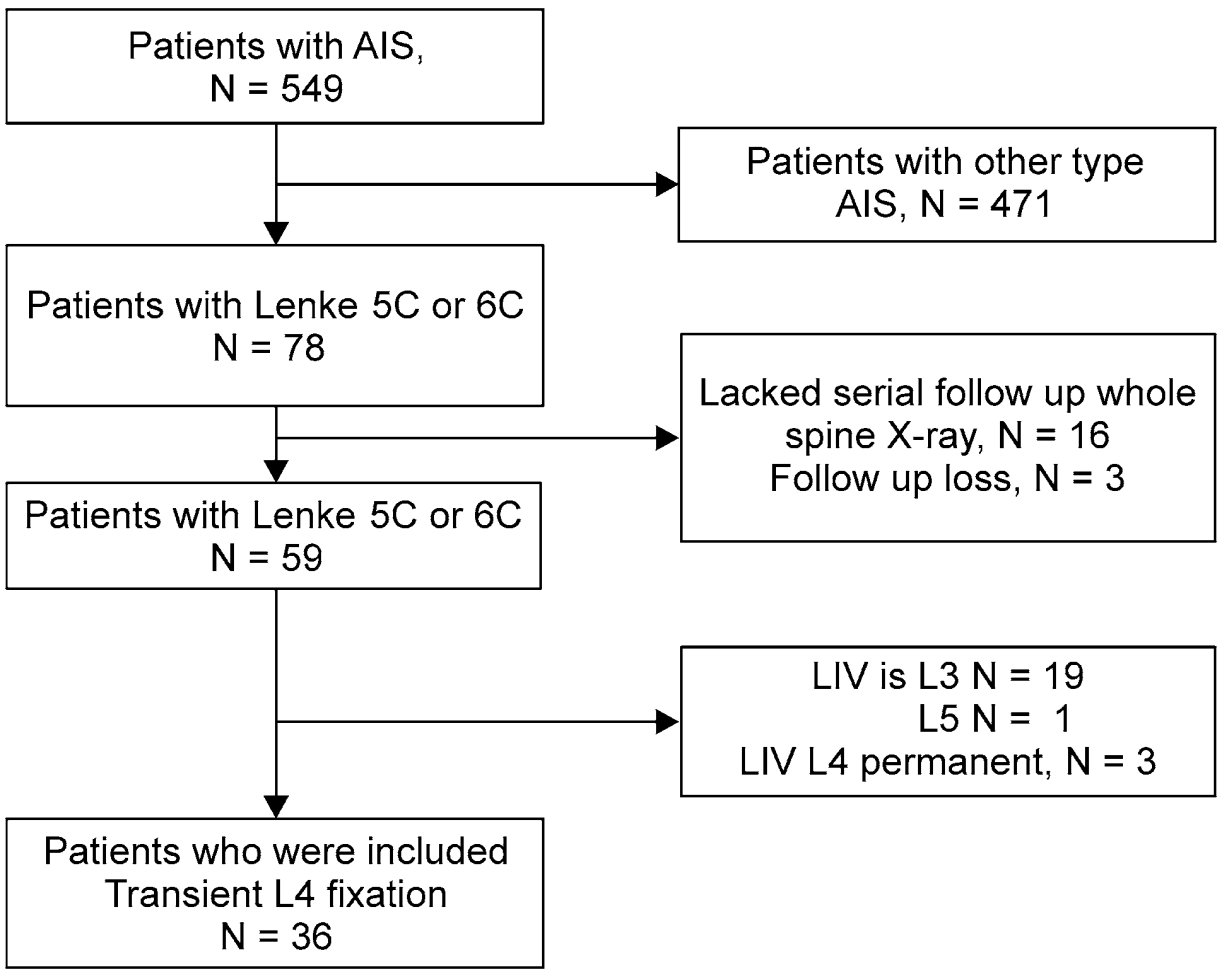
Study flowchart. A total of 549 cases of adolescent idiopathic scoliosis (AIS) that underwent surgical treatment between March 1996 and December 2017 in our hospital were reviewed. Of these, 78 cases were classified as Lenke Type 5C or 6C. Among 78 cases, 16 lacked suitable data regarding serial follow-up whole spine X-ray, and three cases lost to follow-up were excluded from the study. Based on the LIV, 20 cases defined as LIV L3 or L5 were excluded. Further, we excluded three cases that exhibited permanent fixation of L4. Overall, 36 Lenke Type 5C or 6C cases treated with L4 transient fixation were analyzed.

**Figure 2:**
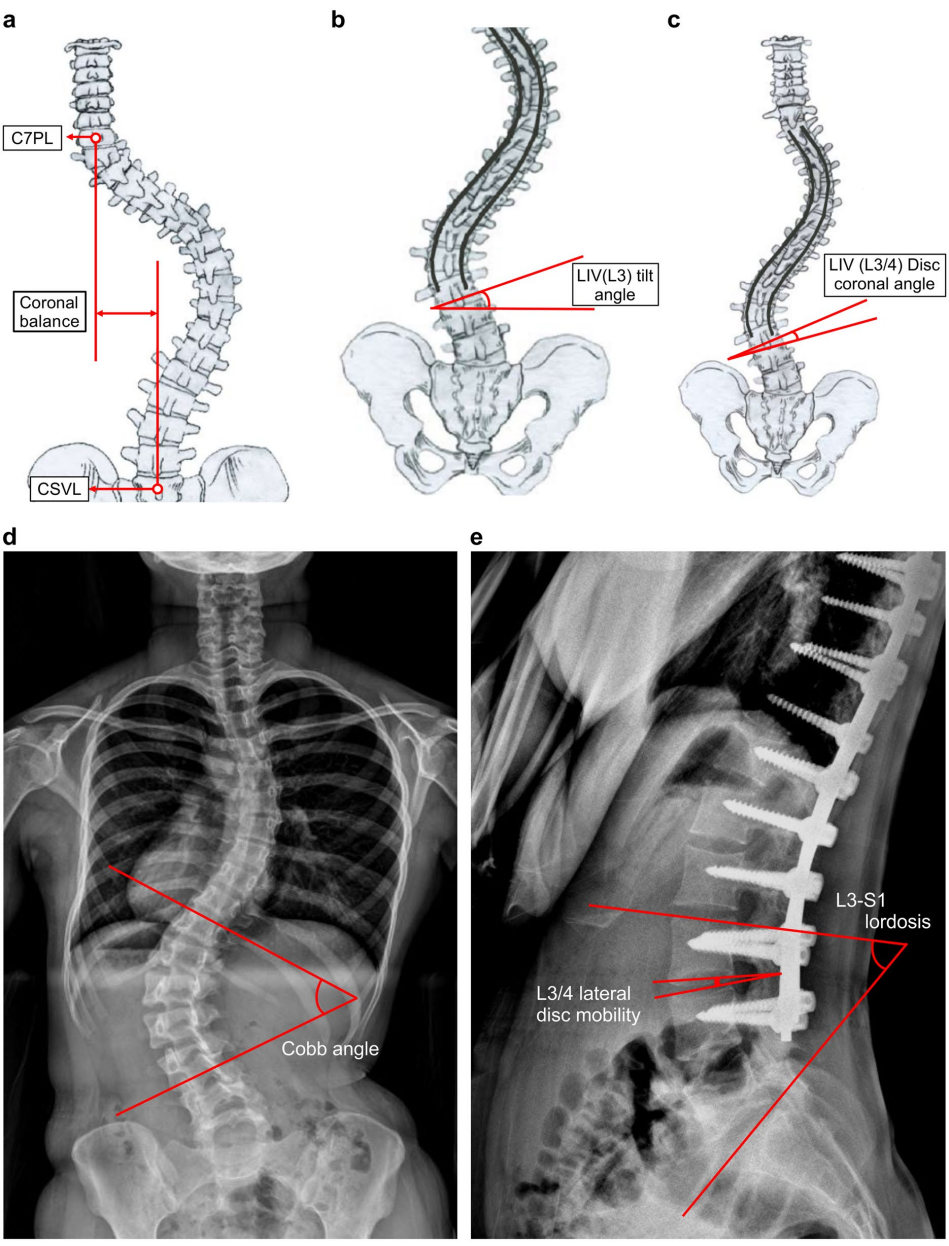
Radiographic parameters measured in the standing whole spine PA, lateral images, and full flexion–extension dynamogram of lumbar spine (**a**) Coronal balance is the distance (mm) between the coronal C7 plumb line and the CSVL. (**b**) The LIV (L3) tilting angle is the angle between the line measured on the inferior endplate of L3 and the horizontal line. (**c**) The LIV (L3/4) disc coronal angle was measured from lines between the L3 lower endplate and the L4 upper endplate. (**d**) The Cobb angle is the angle between the upper and lower end vertebra. (**e**) L3-S1 lordosis is indicated as the line measured on the superior endplate of the L3 and S1 regions. The L3/4 lateral disc mobility is the angle range of the L3/4 disc in full flexion–extension dynamogram.

Thirty-six patients were included in the study (29 females and seven males) (Fig. 1). The average age at surgery was 16.6 ± 2.4 years (Fig. 2). The average skeletal growth was Risser stage 4.6 ± 0.1. The average follow-up time since index surgery was 5.1 ± 1.8 years, which was at least > 2 years for all patients. The interval between the index surgery and transient L4 screw removal surgery was 1.3 ± 0.89 years. There were nine patients with Lenke type 5C and 27 patients with Lenke type 6C. According to the Lenke type, the upper instrumented vertebrae was decided. For the lower end vertebrae in the whole spine standing AP X-ray, L3 was 28 and L4 was 8 (Table 1). Assessment of intra- and inter-observer reliability showed excellent agreement for all investigated radiologic parameters (ICC 0.743–0.812).

**Table 1:** General demographics of patients in this study.

Demographics	Patients with lenke 5C or 6C scoliosis
Sex (M:F)	7:29
Age at first surgery (year)	16.6 ± 2.4 (12.4–20.2)
Risser stage at first surgery	4.6 ± 0.1 (1–5)
Follow-up duration (year)	5.1 ± 1.8 (2.2–9.7)
Period between the first and second surgery (year)	1.3 ± 0.89 (0.3–3.4)
Lenke type (5C:6C)	9:27
Upper instrumented vertebrae (n)	T2(17), T3(5), T4(5), T6(3), T9(6)
Lower end vertebrae (n)	L3(28), L4(8)

Data are presented as Mean ± SD/SE (Range).

### Radiologic and functional parameters

The mean preoperative Cobb angle for the thoracolumbar/lumbar region was 50.2° ± 7.8°. It was decreased to 11.7 ± 4.6° after correction surgery. As shown in Table 2, all radiologic parameters significantly decreased after the first surgery (p < 0.05), which indicated suitable correction of idiopathic scoliosis. After the second surgery involving L4 pedicle screw removal, the corrected Cobb angle was maintained. Furthermore, the procedure did not worsen LIV (L3) tilting or LIV (L3/4) coronal disc angles. Subsequently, coronal balance was maintained (Table 2).

**Table 2:** Comparison of radiographic measurements from baseline, before the second surgery, and at the last follow-up after the second surgery.

Radiologic parameters	Baseline	Before the second surgery	At the last follow-up	P-value*	P-value**
TL/L Cobb angle (^o^)	50.2 ± 7.8 (42.8–64.9)	11.7 ± 4.6 (5.4–19.3)	12.5 ± 4.3 (6.9–20.5)	< 0.01	0.446
Thoracic Cobb angle (^o^)	36.3 ± 13.8 (7.6–72.9)	7.8 ± 3.3 (1.0–14.7)	7.7 ± 3.4 (3.6–17.5)	< 0.01	0.629
Shoulder balance (CHD, mm)	10.2 ± 6.4 (0.4–27.4)	5.6 ± 3.1 (0.1–15.0)	5.3 ± 2.9 (1.2–14.4)	< 0.01	0.687
Sagittal vertical axis (mm)	0.1 ± 20.1 (-39.3–39.8)	5.5 ± 13.4 (−22.4–64.6)	1.9 ± 8.0 (−8.0–14.5)	0.245	0.145
Coronal balance (mm)	19.6 ± 7.9 (10.7–45.6)	9.3 ± 5.3 (3.8–26.9)	8.9 ± 3.6 (3.1–15.7)	< 0.01	0.271
LIV(L3) tilting angle (^o^)	24.3 ± 4.9 (10.5–32.7)	4.4 ± 2.1 (1.1–7.4)	4.7 ± 2.2 (0.5–8.9)	< 0.01	0.304
LIV(L3/4) coronal disc angle (^o^)	4.4 ± 3.4 (0.4–12.5)	1.9 ± 1.3 (0.1–4.3)	2.5 ± 1.6 (0.5–9.5)	< 0.01	0.265
L3/S1 lordosis (^o^)	42.4 ± 6.2 (34.7–52.3)	35.6 ± 6.3 (24.4–43.1)	39.3 ± 4.3 (30.2–45.7)	< 0.01	**0.03**
L3/4 lateral disc mobility (^o^)	6.6 ± 1.8 (4.1–9.2)	NA	4.3 ± 1.7 (2.4–6.5)	** < 0.01**^**§**^	

Data are presented as Mean ± SD/SE (Range). *: p-value from paired t-test comparing values at baseline with those before the second surgery. **: p-value from paired t-test comparing values before the second surgery with those at the last follow-up. ^§^: p-value from paired t-test comparing values at baseline with those at the last follow-up. NA; not accessible. TL/L: Thoracolumbar/lumbar; CHD: Coracoid head difference.

**Figure 3:**
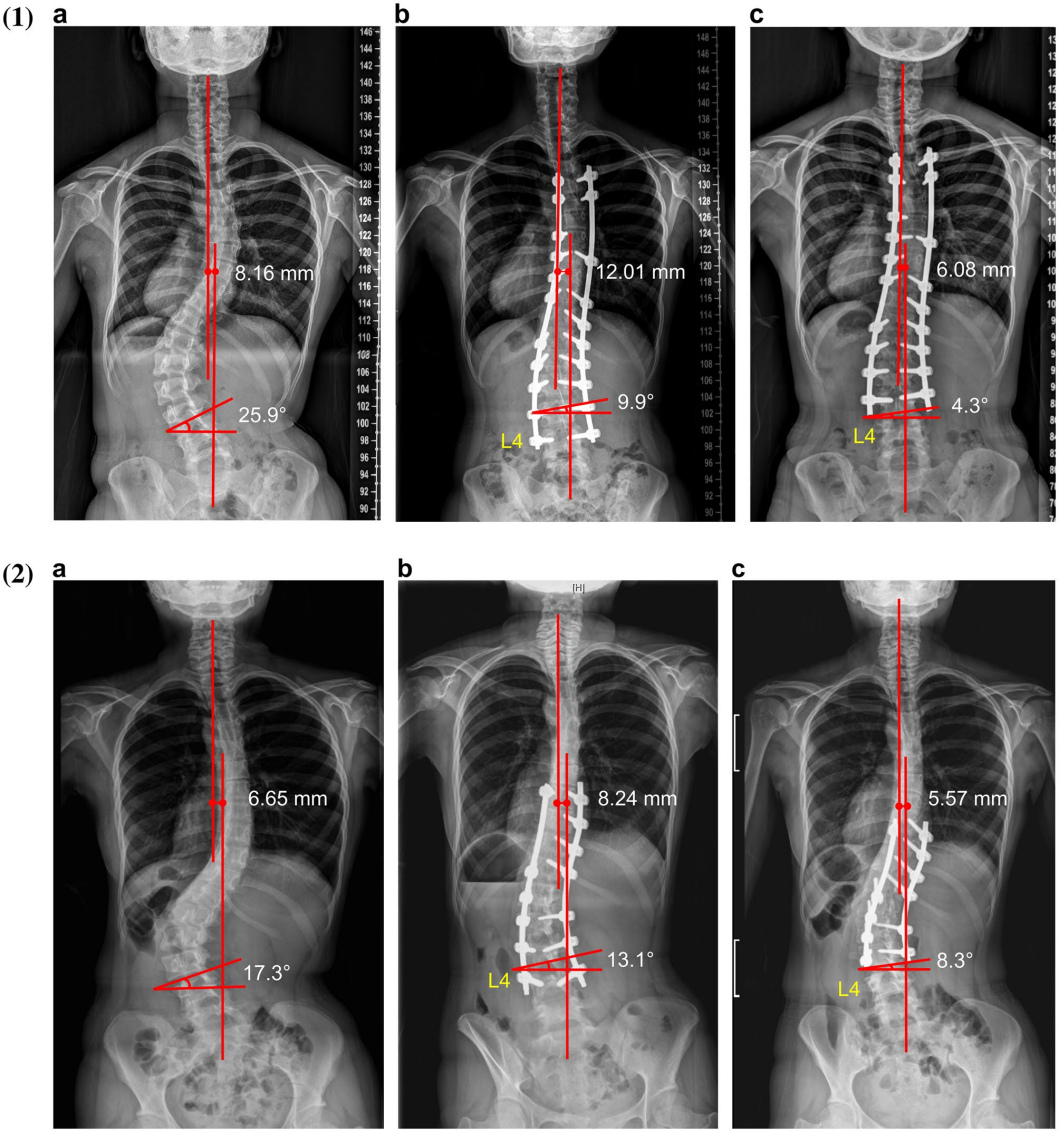
(1) Serial whole spine standing X-ray images, representing a 16-year-old Lenke Type 6C patient (**a**) Preoperative LIV (L3) tilt angle is 25.9°, and the L3 displays a second-degree Nash-Moe rotation. Coronal balance is 8.16 mm. (**b**) After the first surgery (transient fixation) up to L4, the LIV (L3) tilt angle improves to 9.9^°^, the L3 normal rotation improves, and the coronal balance slightly worsens to 12.01 mm. (**c**) The second surgery (L4 transient screw removal) was performed 6 months after the first surgery. In the final follow-up radiograph (2 years and 3 months after the first surgery), the LIV (L3) tilt angle is 4.3°, L3 normal rotation is maintained, and coronal balance is 6.08 mm. (2) Serial whole spine standing X-ray images, representing a 17-year-old Lenke Type 5C patient (**a**) The preoperative LIV (L3) tilt angle is 17.3°, and the L3 displays a second-degree Nash-Moe rotation. Coronal balance is 6.65 mm. (**b**) After the first surgery (transient fixation up to L4), the LIV (L3) tilt angle improves to 13.1^°^, the L3 normal rotation improves, and the coronal balance slightly worsens to 8.24 mm. (**c**) The second surgery (L4 transient screw removal) was performed 11 months after the first surgery. In the final follow-up radiograph (3 years after the first surgery), after the second surgery, the LIV (L3) tilt angle is 10.3^o^, and coronal balance is 5.57 mm.

**Figure 4:**
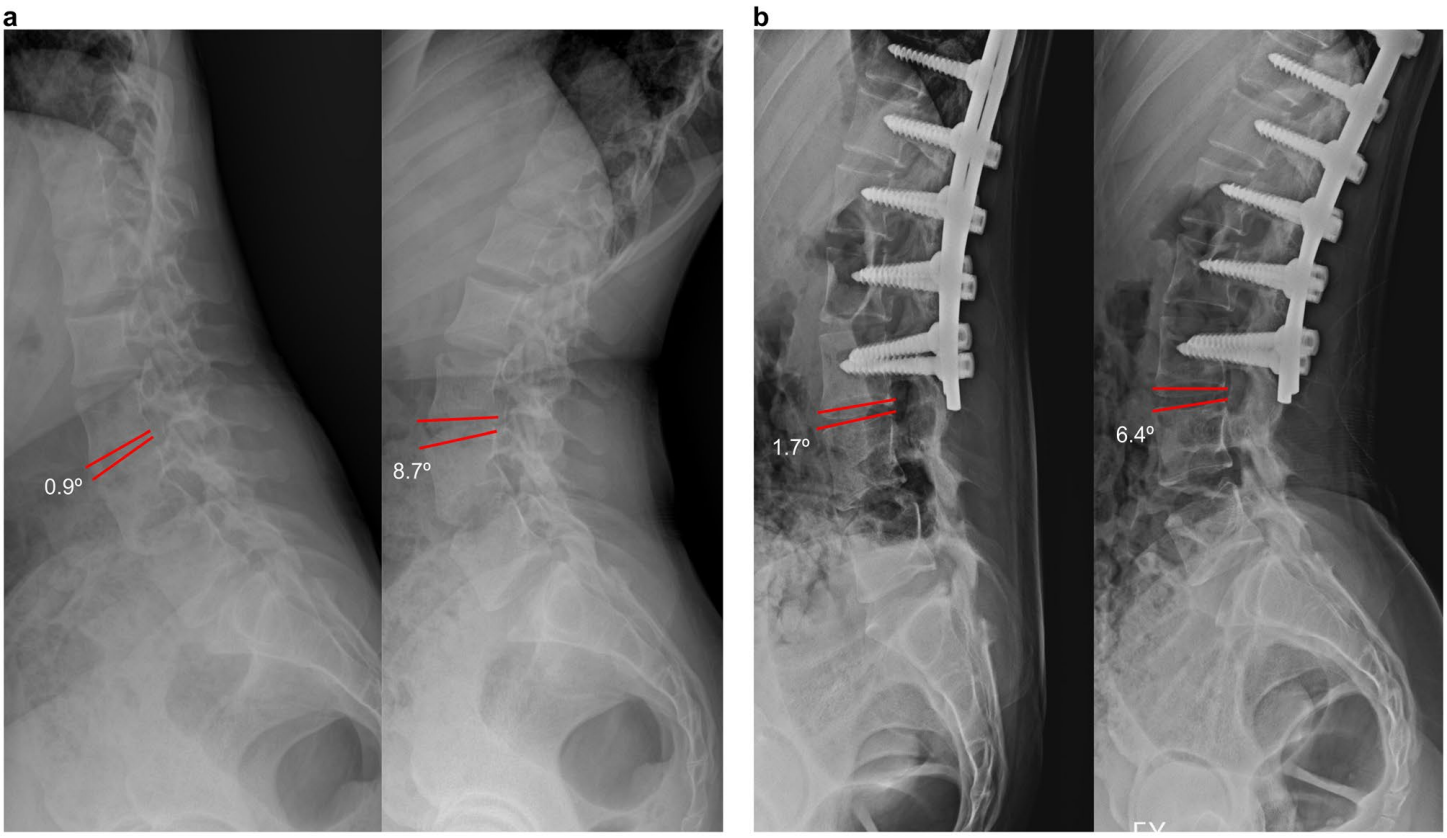
L3/4 lateral disc mobility in full flexion–extension dynamogram of the same patient shown in Fig. 3–1. (**a**)The preoperative L3/4 lateral disc mobility is 7.8°. (**b**) The L3/4 lateral disc mobility of 4.7° at the last follow-up, after the second surgery.

**Figure 5:**
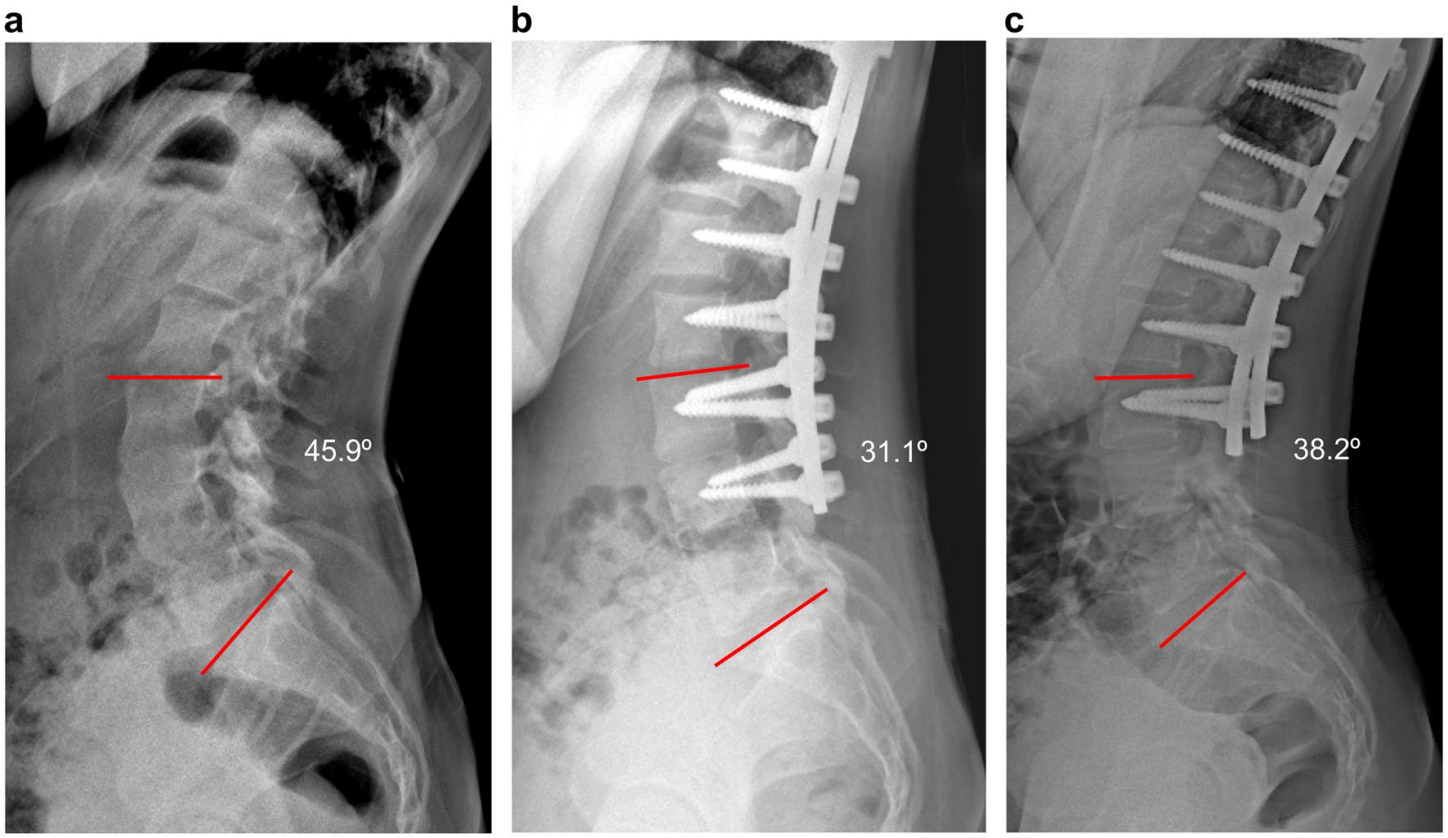
L3/S1 lordosis in whole spine lateral images of the same patient shown in Fig. 3–1 (**a**) Preoperative L3-S1 lordosis is 45.9°. (**b**) After the first surgery (L4 transient fixation), the L3-S1 lordosis angle is 31.1°. (**c**) After the second surgery (final follow-up), the L3-S1 lordosis angle is 38.2°; the lumbar lordosis is slightly increased.

The preoperative L3/4 lateral disc mobility was 6.6 ± 1.8°. After the first surgery, including the fixation with L4 pedicle screws, the L3/4 lateral disc motion angle could not be measured because the L4 vertebra was fixed. However, at the final follow-up after the second surgery, despite the angle being significantly reduced to 4.3 ± 1.7° (p < 0.01 compared to preoperative values), L3/4 lateral disc mobility was eventually restored after the removal of the L4 transient pedicle screws. (Table 2). The representative cases of Lenke 6C and 5C in this study are shown in Fig. 3-1 and -2. Preoperative lumbar lordosis between L3-S1 was 42.4° ± 6.2°. After the first surgery, lumbar lordosis was significantly reduced to 35.6° ± 6.3° (p < 0.01). However, after the removal of transient L4 pedicle screws, L3/S1 lumbar lordosis was slightly increased to 39.3° ± 4.3° (p = 0.03, compared to the angle before the second surgery). (Table 2) Fig. 4 shows L3/4 lateral disc mobility in full flexion–extension dynamogram of the same patient shown in Fig. 3-1 and Fig. 5 shows L3/S1 lordosis in whole spine lateral images of the same patient shown in Fig. 3-1.

Among the SRS-22 scores consisting of four domains (i.e., pain, self-image, function, and mental health), only self-image improved (p < 0.01) after the first surgery. While, following the removal of the L4 transient screws, patients reported better scores only in the function domain of the SRS-22 (p = 0.04) (Table 3).

**Table 3:** Comparison of SRS-22 results at baseline, before the second surgery, and at the last follow-up after the second surgery.

SRS-22 domain	Baseline	Before the second surgery	At the last follow-up	P-value*	P-value**
Pain	3.8 ± 0.57 (3–5)	3.9 ± 0.59 (3–5)	4.1 ± 0.41 (3–5)	0.31	0.12
Self-image	3.5 ± 0.49 (3–4)	4.3 ± 0.55 (3–5)	4.5 ± 0.5 (4–5)	** < 0.01**	0.13
Function	4.0 ± 0.36 (3–5)	4.1 ± 0.36 (3–5)	4.3 ± 0.44 (4–5)	0.45	**0.04**
Mental	4.2 ± 0.41 (4–5)	4.3 ± 0.55 (3–5)	4.4 ± 0.49 (4–5)	0.27	0.28

*: P-value from paired t-test comparing the values of baseline with ones before the second surgery. **: p-value from paired t-test comparing the values before the second surgery with those at the last follow-up after the second surgery. The second surgery: removal of L4 transient pedicle screws.

### Data of the second surgery

In the earlier L4 transient fixation procedures, we had recommended the removal of the L4 screws about 1 or 2 years following the first surgery due to the possibility of the worsening of the scoliotic curve. However, we did not experience worsening of cases; we tried shortening the interval period for the restoration of segment lumbar motion to 3 months after the first surgery. There were no cases of correction loss or the adding-on phenomenon requiring revisional surgery. However, for one patient, the breakage of L4 pedicle screws was identified 2 years after the first surgery. In contrast, one patient who had the L4 screws removed 4-months after the first surgery exhibited an increase in the LIV (L3/4) coronal disc angle to 9.5°. In the first case, we just removed the broken screws and cut the rods. In the latter case, the LIV (L3/4) coronal angle increased by 9.5°, which was not considered as a marked loss of coronal balance. Therefore, we observed the patient, and it was maintained without more aggravation. The average period for the second surgery, L4 screws removal, was 1.2 ± 0.3 h, and the estimated blood loss was 102 ± 23.5 ml. No deep wound infections were noted in the patients during the first and second surgery (data not shown).

## Discussion

Despite advances in scoliosis surgeries, there is still a major controversy regarding the distal fusion level for idiopathic scoliosis surgery ^5,7–14^. A 40-year follow-up study by Lander et al. ^12^ indicated that there was a higher risk of additional surgery and poorer functional outcome after L4 or below L4 fixation in idiopathic scoliosis surgery. In a 19-year follow-up study by Bartie et al. ^15^, pain intensity for non-scoliosis controls was equivalent to L2 and L3 fusion patients; L4 fusion patients experienced increased levels of pain. However, a small study with 30 subjects and a 2-year follow-up reported no differences in clinical results between the L3 fixed group and the L4 fixed group ^16^. Despite these issues, many researchers have attempted to preserve the L4 vertebra by setting the L3 vertebra as the LIV in patients with Lenke 5C or 6C type scoliosis ^17^. However, we often encounter cases that partially fulfill the criteria for L3 LIV. In our study, if one of the L3 selection criteria was violated, then L4 transient fixation was performed. Therefore, 36 cases (61%) comprised the transient L4 group.

The correction rate of the main thoracolumbar/lumbar curve because of our transient L4 fixation surgery was 76.6%, which was similar to previous studies ^9,18^. After removal of the L4 screws in the second surgery, correction loss did not occur. In our study, the coronal balance correction ratio from transient L4 fixation procedures was approximately 54.6%. Comparing this ratio with the one from Ding et al.'s study ^16^, this correction ratio was arithmetically superior compared to the 50% (6.5 ± 5.5/13.0 ± 9.0 mm) correction ratio observed when L3 was fixed. However, it was inferior to 65% (7.2 ± 6.8/21.1 ± 11.1 mm) when L4 was fixed. Recently, Shu et al. ^11^ reported a coronal balance correction ratio of 22% in a distal adding-on group when the LIV presented L3 in Lenke Type 5C scoliosis; however, it was 57% in patients not exhibiting adding-on phenomenon ^19^. Considering results from previous studies, we could assume that the transient L4 fixation might correct coronal balance effectively even without fusion up to the L4 and might lower the potential for adding-on phenomenon.

The LIV tilting angle and LIV coronal disc angle are important radiographic factors for evaluating the results of lumbar type C scoliosis surgery. Kim et al. ^9^ reported an increased LIV tilting angle of 11.0 ± 5.3° when the LIV was L3 in patients with L3 rotation above those of Nash-Moe Grade II and the L3 did not cross the CSVL on the bending film. Whereas, in the group in which L3 crosses the mid-sacral line without rotation inferior to grade II, the LIV tilting angle was 3.3 ± 2.9°. The former group satisfied two of the three criteria for transient L4 fixation in this study. Similarly, Shu et al. ^11^ reported worse results with a LIV tilting angle of 11.0 ± 3.5° in patients with adding-on phenomenon than the group without adding-on phenomenon with LIV (L3) in Lenke Type 5C scoliosis. In this study, the final LIV (L3) tilting angle was 4.7 ± 2.2°. The LIV (L3/4) coronal disc angle at the final follow -up (2.5 ± 1.6°) was not significantly different from values after first surgery (1.9 ± 1.3°, p = 0.265). Additionally, similar to the LIV tilting angle, Chang et al. ^7^ reported worsening of LIV (L3/4) coronal disc angles of 3.8 ± 2.2° in the L3 rotation group than in Nash-Moe Grade II or in cases where L3 did not cross the CSVL on bending film compared to the group of L3 cases crossing the mid-sacral line with no rotation of below grade II (2.7 ± 2.4°, p = 0.031). These results indicated that transient L4 fixation had the advantages of maintaining the LIV tilting angle and LIV coronal disc angle even in patients presenting severe grade of L3 rotation and translation without adding-on phenomenon.

When performing L4 transient fixation, there were two major concerns. First, if the fixed period is more than a few years, the L3/4 disc may or may not move after L4 screw removal. Second, however, if the fixed period is only a few months, the corrected deformity might reappear. After the second surgery, the L3/4 lateral disc mobility was maintained at 65% of the original value, and the L3/S1 lordosis slightly improved after the removal of L4 screws, suggesting that a transient fixation of the L3/4 facets maintains function in most cases without correction loss. Therefore, removing the L4 screws after a certain period had no impact on the results of the lumbar C scoliosis surgery. Nonetheless, the screw broke 2 years after the first surgery in one case, and the LIV (L3/4) coronal angle increased in one case in which the transient L4 screws were removed 4 months after the first surgery. These results suggested that the timing for removing the screw requires careful attention.

Similar to other studies ^20–24^, SRS-22 analysis revealed a statistically significant difference in the self-image domain comparing pre- and post-operation evaluations. However, the change in self-image domain was not large, which may be due to a large proportion of patients indicating a maximum score in the questionnaire. Interestingly, despite minimal changes, the function analysis from the SRS-22 indicated a statistically significant difference between the first and second surgery. This main difference was due to one question which assesses back function limitations. According to Carreon et al., ^25^ the minimum clinically important difference in function domain in SRS-22 scores after surgical correction of AIS is 0.08. Therefore, the statistically significant increase of 0.2 in the function domain in this study is sufficient to produce a significant clinical improvement.

This study has a few limitations. First, it was a retrospective study. Second, the patients included in this study were relatively small. Third, the optimal timing of L4 transient screw removal was not adjusted because this study only investigated the data retrospectively, and the interval became shorter when we applied the surgical method. However, it should be noted that the new concept of the surgical method could be applied in patients with Lenke Type 5C and 6C scoliosis. Fourth, the real effect of transient L4 fixation on L3/4 facet joints was not evaluated because postoperative computed tomography scan was not performed routinely in all patients. With more patients and a prospective study design, the optimal interval and real effect on facet joints of transient L4 screw fixation is recommended for future evaluation.

In conclusion, the L4 transient fixation surgery method preserves L3/4 disc motion and increases functional outcome while obtaining equally improved correction and coronal balance outcomes in Lenke Type 5C and 6C scoliosis cases, which did not fully satisfy the L3 LIV criteria.

## Methods

### Study design and population

This study was approved by the institutional review board of Gangnam severance hospital (IRB No. 3–2020-0119). All investigations were carried out in accordance with relevant guidelines and regulations of our licensing committee. The requirement of informed consent was waived by the institutional review board of Gangnam severance hospital because this was a retrospective study.

We reviewed 549 cases of patients with adolescent idiopathic scoliosis (AIS) who underwent surgical treatment between March 1996 and December 2017 in our hospital. Of these, 78 cases were classified as Lenke Type 5C or 6C. Among 78 cases, 16 cases lacking suitable data relative to serial follow-up whole spine X-ray, and three cases lost follow-up were excluded from the study. Based on the lowest instrumented vertebra (LIV), 20 cases in which LIV was L3 or L5 were excluded. We also excluded three cases with a permanent fixation of L4. Overall, 36 Lenke 5C or 6C cases treated with L4 transient fixation were analyzed (Fig. 1). Their follow-up period was > 2 years.

### Treatments and data collection

All 36 patients underwent instrumented spinal fusion using CD Horizon spinal instrumentation (Medtronic, Memphis, TN, USA). We used a slightly modified Lenke criteria set ^6^ to identify the LIV. The modified criteria required: (1) L3 tilt of < 25°, (2) L3 rotation less than Nash-Moe Grade II on bending film, and (3) L3 crossing the CSVL on bending film ^9^. If all three criteria were satisfied, L3 was determined as the distal LIV. If none of the criteria were present, L4 or L5 permanent fixation was performed. When one or two of the three criteria were not satisfied, L4 transient fixation was performed. The bilateral facet capsule of L3-4 was preserved during the exposure, and heads of L4 pedicle screws were placed slightly above the capsule, which was meant not to violate the capsule and facet joints. At the second surgery, a small incision was made on the distal part of the preoperative scar, and L4 pedicle screws were exposed. The caps of pedicle screws were untightened. The distal rods were cut using a rod cutter and removed. Then, bilateral L4 pedicle screws were removed.

In the early phase of this study, because we removed the transient L4 screws 1 or 2 years later, we recommended the application of a hard brace for 3 months after the first surgery and a return to low-level sports activity, such as jogging. After the second surgery, we applied the hard brace only for 2 weeks and did not limit activity. In the later phase of this study, we recommended only activities of daily living with the 3-month hard brace application after the first surgery. Then, we removed the L4 transient screws. Afterward, we extended the application of the hard brace for an additional 2 weeks. After that period, we gradually permitted low-level sports activities.

The patients’ age and Risser stage on preoperative pelvis anteroposterior (AP) X-ray were investigated. All radiologic parameters were measured at three-time points; prior to the first surgery, before the second surgery of L4 screw removal, and at the final follow-up after removal of the L4 screw. Consequential radiologic changes were assessed after the removal of transient L4 pedicle screws. The following radiographic parameters were investigated; (1) Cobb angle (Thoracolumbar/lumbar and thoracic), (2) shoulder balance (coracoid head difference (CHD)), (3) sagittal vertical axis, (4) coronal balance, (5) LIV (L3) tilting angle, (6) LIV (L3/4) coronal disc angle on standing whole spine posteroanterior (PA) images, (7) L3-S1 lordosis on standing whole spine lateral images, and (8) L3/4 lateral disc mobility on full flexion–extension dynamogram of the lumbar spine. Methods used for the measurement of angles are shown in Fig. 2. To evaluate the intra- and inter-observer reliability of radiographic parameters, two spine surgeons independently measured the radiologic parameters twice, over at least a 2-week interval. They were external to this study, and all clinical data were blinded to them.

Data from SRS-22 scores were also collected at the three-time points to assess patient-centered outcomes, including pain, self-image, function, and mental health.

### Statistical analysis

Radiographic parameters and each SRS-22 domain were compared by calculating the mean and standard deviation (SD). A paired t-test was used to compare radiographic parameters and SRS-22 scores between those at the baseline, after the first surgery, and after the second surgery. Intra- and inter-observer agreements were assessed using the intraclass correlation coefficient (ICC). The ICC with 95% confidence interval (CI) was also calculated, comparing the mean of all two trials for the two observers. ICCs < 0.40 indicate poor; 0.40–0.75, fair or good; and 0.75–1.00, excellent reliability. A p-value < 0.05 was considered to be statistically significant. SAS v9.3 (SAS Institute, Cary, NC, USA) was used for the statistical analyses.
